# Bladder Microbiome in the Context of Urological Disorders—Is There a Biomarker Potential for Interstitial Cystitis?

**DOI:** 10.3390/diagnostics12020281

**Published:** 2022-01-22

**Authors:** Thomas Bschleipfer, Isabell Karl

**Affiliations:** Klinik für Urologie, Andrologie und Kinderurologie, Interdisziplinäres Kontinenz- und Beckenbodenzentrum, Zentrum für Interstitielle Zystitis (IC) und Beckenschmerz, Klinikum Weiden/Kliniken Nordoberpfalz AG, Söllnerstr. 16, 92637 Weiden in der Oberpfalz, Germany; isabellkarl@gmx.de

**Keywords:** urinary microbiota, next-generation sequencing, enhanced quantitative urine culture (EQUC), 16S rRNA sequencing, Hunner lesion

## Abstract

Since the development of modern cultivation and sequencing techniques, the human microbiome has increasingly become the focus of scientific attention. Even in the bladder, long considered to be a sterile niche, a highly variable and complex microbial colonization has now been demonstrated. Especially in the context of diseases such as interstitial cystitis, whose etiopathogenesis is largely unknown, and whose diagnosis is based on a process of exclusion of confusable diseases, science hopes to gain far-reaching insights for etiology and diagnosis, including the identification of potential biomarkers. While for functional disorders such as urge urinary incontinence and overactive bladder syndrome, initial associations have been demonstrated between reduced microbial diversity and increased symptomatology, as well as shifts in the abundance of specific microorganisms such as *Lactobacillus* or *Proteus*, studies in interstitial cystitis show conflicting results and have failed to identify a putative organism or urotype that clearly distinguishes the urinary microbiome of patients with IC/BPS from that of healthy controls. At the present time, therefore, the new insights into the bladder microbiome and its potential influence on urologic disease cannot yet be used in the context of elucidating possible etiopathogenetic causes, as well as in the use of a biomarker for diagnostic or prognostic purposes. Further studies should focus primarily on uniform procedures and detection methods to achieve better comparability of results and increase the likelihood of detecting hidden patterns.

## 1. Introduction

Recently, advances in molecular biology techniques and culture methods have made the study of the human microbiome increasingly important and have facilitated the definition of specific microbiomes in several physical areas of the human body. In a healthy state, the humans hosts a variety of microorganisms, including bacteria, fungi, viruses and protozoa [1,2]. It is now widely recognized and appreciated that there are associations between specific disease states and alterations in the microbiome and that the microbiome controls various physiological processes in both health and disease [2,3,4,5,6]. However, although the role of specific disease states with variations in cutaneous, gastric, colon, and gut microbiomes has been well explored by a large number of studies, the urinary tract was considered sterile for a long time [7,8,9]. Due to recent findings, however, it is now clear that even in a healthy state, the bladder does not represent a sterile niche [10,11,12], a point which has fundamentally changed our perspective toward the role of bacteria in the urinary tract and our perception of the health of the genitourinary system [13]. Although exact functions of the bladder microbiome remain to be elucidated, the results of many studies suggest that alterations in the urinary microbiome, both in abundance of specific organisms and/or overall diversity, may be triggers, amplifiers, or possible connecting factors for various functional disorders of the lower urinary tract [7,14,15].

Interstitial cystitis/bladder pain syndrome (IC/BPS) is a disease of unknown etiology, presenting symptoms of intermittent or chronic pelvic pain/bladder pain and lower urinary tract symptoms, such as frequency and urgency in the absence, respectively, without the verification of a typical urinary tract infection [16,17]. Measured in terms of the number of cases, interstitial cystitis (IC) by definition belongs to the rarer diseases (orphan diseases) in Europe, with a prevalence of <5/10,000 inhabitants. With a prevalence of 52–500/100,000, female IC/BPS patients are affected about nine times more often than male patients, where IC/BPS occurs in about 8–41/100,000 persons [18,19]. The symptoms themselves may be associated with or without disease-typical cystoscopically and/or histologically detectable changes in the urinary bladder. In the Hunner type, clear lesions can be detected by cystoscopy, whereas in the much more common non-Hunner type, such clearly detectable changes are absent [20,21].

To date, the pathogenesis and etiology of IC/BPS is largely unknown, and a variety of confounding conditions make it a diagnosis of exclusion [16]. Against proposed structural alterations in the bladder wall’s glycosaminoglycan (GAG) layer and urothelial permeability, an upregulation of neuroendocrine pathways, autoimmunity, a central dysregulation of bladder sensitivity with alterations in the central nervous system (particularly the sympathetic system) and sensory innervation, a cytokine-mediated inflammatory reaction and mast cell activation [16,22,23,24], in this context, even a microbiome-based etiology has never been ruled out as a mechanism in IC/BPS. Consequently, within the last years, an increasing number of studies have been trying to detect specific patterns or even specific bacteria or microorganisms that could be causative for the symptomatology of IC/BPS disease and possibly receive importance as biomarkers in diagnosis.

Therefore, in addition to a general overview of previous findings regarding the human bladder microbiome and possible associations with lower urinary tract symptomatology, in this narrative review, we particularly aim to present a comprehensive summary of previous findings regarding changes in the microbiome, specifically in IC/BPS patients, and to evaluate its potential suitability as a biomarker for diagnostic purposes.

## 2. The Human Urinary Microbiome

### 2.1. The Modern Way of Urinary Microbiome Determination

Standard urine cultures are designed to detect only the most common, fast-growing, aerobic uropathogens, such as *Escherichia coli* [11]. Anaerobic bacteria, bacteria that require specific nutrients and grow slowly, are present in low numbers (<103 colony-forming units per milliliter), or are embedded within biofilms. However, they are not detectable by conventional standard urine cultures [25]. Only by applying culture-independent metagenomic analysis using next-generation sequencing (NGS) or by enhanced quantitative urine culture (EQUC) with mass spectrometry a more comprehensive assessment of microorganisms within the bladder and rapid progress in urinary microbiome knowledge became possible [10,11,12].

NGS techniques can be based on both amplicon sequencing, and shotgun sequencing. Amplicon sequencing consists of PCR-based analysis focused on marker genes, such as the 16S rRNA subunit, to determine the evolutionary distance between different bacterial species and highly conserved interregional sequences for primer design [11,26]. As a highly conserved component of the transcription machinery of all DNA-based life forms [27], and large enough with sufficient interspecific polymorphisms to provide distinguishing and statistically valid measurements [28], the 16S rRNA gene is exceedingly suited as a target gene for sequencing samples containing a multiplicity of different species. Furthermore, the 16S rRNA gene is universal in bacteria; thus, relationships can be measured among all bacteria, and taxonomic and evolutionary associations could reliably be revealed [29,30]. Shotgun sequencing, in contrast, is based on whole-microbiome sequencing in a wide variety of samples. In addition to bacterial components, viruses or fungi can be recorded [31].

To overcome one limitation of the DNA sequencing-based methods, namely, its inability to show the viability of the identified bacteria, expanded quantitative urine culture (EQUC, [32,33]) has additionally been developed. With this modified culture protocol involving plating out larger and variable volumes of urine, incubation under different atmospheric conditions (including, e.g., aerobic and anaerobic conditions) and longer incubation times [32,33], a large spectrum of organisms identified in urine by 16S rRNA gene sequencing could be cultured. With up to 92% of species undetectable by standard urine culture and a 80% agreement between cultured genera and sequenced bacterial DNA [10], this method offers another excellent basis for microbiome studies.

### 2.2. Complex and Variable: The Healthy Human Urinary Microbiome

With the help of new and state-of-the-art techniques for the detection and characterization of the microbial colonization of the human body, the rejection of the pre-established concept of sterility in the urinary bladder has been reaffirmed. Although the microbiota in urine is less abundant (e.g.,~1/10 colony-forming unit (CFU)/mL in comparison to faces [34]) and less diverse than the microbiota in other sites of the body, a wide variety of microorganisms and bacteria have now been described for the urine microbiome [35]. Many of the taxa identified in the healthy urine microbiome include species commonly known as slow-growing microorganisms, which demand specific conditions of their environment [12,15]. They belong predominantly to the five major phyla: *Firmicutes*, *Bacteroidetes*, *Actinobacteria*, *Fusobacteria*, and *Proteobacteria* [36], frequently including the genera *Lactobacillus*, *Corynebacterium*, *Prevotella*, *Gardnerella*, *Staphylococcus*, *Actinomyces*, and *Streptococcus* both in men and women [10,34,35,37,38,39,40,41].

However, such a crudely described core microbiome does not begin to encompass the true individuality, complexity and variability in both abundance and diversity that could be detected for the human bladder microbiota. For example, one study was able to report up to 321 different genera that could be detected in samples from culture-negative patients [12]; however, in the vast majority of studies, significantly lower numbers of different species are detected. Furthermore, several studies have indicated the urinary microbiome to be different between men and women [35,37,40,42,43]. In general, the microbiome diversity of females seems to be more heterogeneous, with an increased occurrence of *Actinobacteria* and *Bacteroidetes* phyla [35,40]. In contrast, another study showed no significant differences for alpha-diversity (intrasample) but did for beta-diversity (intersample) between males and females [42]. Compared on a genus level, female samples showed the highest abundance of *Lactobacillus* and *Prevotella*, while in males *Streptococcus*, *Veillonella*, *Staphylococcus*, *Gardnerella*, *Enterobacter* sp., *Neisseria*, *Haemophilus* and *Corynebacterium* dominated [41,42,44]. Additionally, a reduction in relative abundance of *Lactobacillus*, *Bifidobacteria*, *Sneathia*, *Shuttlewothia* or *Bacillus* with increasing age has been shown for females [39,45,46], with a simultaneous increase in *Mobiluncus*, *Oligella* or *Porphyromonas* after entering menopause [45]. *Jonquetella*, *Parvimonas*, *Proteiniphilum* and *Saccharofermentans,* interestingly, have been exclusively identified in men and women over 70 years of age [40].

## 3. The Bladder Microbiome and Urological Disorders

Now that the old dogma of the sterile bladder has finally fallen in recent years, and the presence of a polymicrobial bacterial community is also accepted in the urinary tract, a new and broad field has opened for research. Unlike other areas of the human body, the role of bacteria in the urinary tract in health and urogenital diseases is largely unknown. In this context, several studies in recent years have been carried out to investigate possible correlations between the presence of specific microorganisms or alterations in the composition of the microbiome in the context of a particular disease or symptomatology [7,8,47,48,49].

### 3.1. Altered Bladder Microbiome in UUI and OAB

Despite large variability in diversity and richness among individual samples [49], a significant association between higher symptom severity (as measured by a higher UDI score and a higher percentage of urge incontinence episodes) with a lower microbial diversity was found in patients with urge urinary incontinence (UUI) [34,49]. Although *Lactobacillus* was isolated from both cohorts, healthy women showed more *Lactobacillus* sequences compared to women with UUI. In addition, there are differences at the species level, with *Lactobacillus gasseri* more frequently detected in the UUI cohort and *Lactobacillus crispatus* most frequently detected in the control group [34,50,51]. Both species are also regularly found in the vaginal microbiota, and both are considered protective in this niche. This finding suggests that these two species play distinct roles in the bladder [34]. Specific bacteria previously associated with UTIs (*Alteromonadaceae* spp., *Stenotrophomonas* spp., *Brevundimonas* spp., *Elizabethkingia* spp. and *Methylobacterium* spp. [52,53,54,55,56]) were also detected in increased abundance in women with UUI [49], suggesting a persistent low-grade infection of bacteria being responsible for the irritative symptoms of UUI, at least for some individuals.

Associations in symptomatology and altered bladder microbiome have also been described in overactive bladder disease (OAB). Based on the weighted UniFrac distance metric, significantly different bacterial communities of OAB and controls could be detected [57]. In the OAB group, 13 genera were underrepresented, namely *Prevotella*, *Dialister*, *Fusobacterium*, *Jonquetella*, *Campylobacter*, *Finegoldia*, *Anaerococcus*, *Lactobacillus*, *Pyramidobacter*, *Ureaplasma*, *Enterococcus*, *Novosphingobium* and *Lactococcus*. In contrast, seven genera were overrepresented in the OAB group, namely *Sneathia*, *Staphylococcus*, *Proteus*, *Helcococcus*, *Gemella*, *Mycoplasma* and *Aerococcus*. In particular, the less frequent detection of *Lactobacillus* DNA in women with OAB [58] and the clustered presence of *Proteus*, a genus with many uropathogenic species [59], were reconfirmed in additionally studies.

### 3.2. Conflicting Evidence for Associations between Alterations in the Bladder Microbiome and IC/BPS

Due to the largely unclear etiopathogenesis, the variability of symptoms and the multiplicity of confusable diseases, the diagnosis of interstitial cystitis has, to date, been based on a process of exclusion [16,17], which makes early recognition and treatment of affected patients difficult. Therefore, the current and initial findings of the bladder microbiome in the context of urologic symptoms, which also occur in interstitial cystitis, have also focused on the possible identification of triggering factors, an etiologic basis, but also on potential new diagnostic, prognostic or predictive biomarkers based on the microbiome in the context of IC/BPS.

Siddiqui and colleagues [60] used a high-throughput sequencing analysis of clean-catched midstream urine from women with interstitial cystitis. Compared to historical controls (data from a previous study [36]), the authors were able to show marked differences in taxonomic composition, richness, and diversity in the microbial profile between the two groups. There was a considerable reduction in the total numbers of genera identified in IC urine (31 genera vs. 45 genera in healthy women), and, indicated by rarefaction analysis, the number of OTUs, Shannon index and inverse Simpson index estimation, a significant decrease in overall richness and ecological diversity in IC/BPS-samples compared to healthy women could be determined. Furthermore, a decrease in abundance of *Prevotella* and *Gardnerella*, evidence of four genera exclusively identified in IC/BPS patients (*Enterococcus*, *Atopobium*, *Proteus* and *Cronobacter*), a group of 17 genera only associated in urine of healthy women and, interestingly, a significant increase in *Lactobacillus* in interstitial cystitis samples suggested a structural shift in the microbiota of IC/BPS-patients.

Abernethy et al. [61] performed a cross-sectional study by sequencing the catheterized urine of 40 participants. Similar to Siddiqui et al. [60], women with interstitial cystitis showed less microbial diversity and fewer distinct operational taxonomic units than those in the control group. Greatly varying from the aforementioned study, however, the researchers found urine from women with interstitial cystitis less likely to contain *Lactobacillus* species, specifically, *L. acidophilus*. In addition, this study has demonstrated associations between the presence of *L. acidophilus* and lower scores on the Interstitial Cystitis Symptoms Index and the Female Genitourinary Pain Index. The extent to which there might be a possible correlation with an already known anti-inflammatory effect of *L. acidophilus* [62,63,64], or whether an overall protective role of *Lactobacillus*, similarly known from the vaginal environment [65], is of significance for the documented patterns, remains elusive.

In a larger study with 181 females with IC/BPS and 182 female control participants enrolled in the National Institutes of Health (NIH) Multidisciplinary Approach to the Study of Chronic Pelvic Pain (MAPP) research network [66], an analysis of the relative abundances could not demonstrate significant differences within the two cohorts in most of the 92 detected species (in 41 genera). Nevertheless, on the species level, a tendency to a greater abundance of *L. johnsonii* and *L. gasseri* in the participants with interstitial cystitis and *L. acidophilus* in healthy controls and, on the genus level, a significant greater relative abundance of *Corynebacterium* *sp*. in controls could be detected. Furthermore, 29 species, including bacteria that are considered potentially uropathogenic (such as *Escherichia coli*, *Proteus mirabilis* and *Pseudomonas aeruginosa*), as well as potentially virulent organisms (such as *Francisella tularensis*, *Mycoplasma hyorhinis*, *Helicobacter hepaticus*, *Clostridium perfringens* and *Candida dubliniensis*), could be exclusively verified in the urine of IC/BPS patients.

Moreover, Xu et al. [67] were able to demonstrate a shift in several genera, with an overrepresentation of *Serratia*, *Brevibacterium*, *Porphyromonas*, and *Citrobacter* and with concomitantly underrepresented *Senegalimassilia*, *Howardella*, *Gemella*, *Dialister*, *Moheibacter*, *Sphingobium*, *Fastidiosipila*, *Megasphaera*, *Thermovum*, *Mycobacterium* and *Atopobium* in samples of the IC/BPS cohort. At the family level, *Lactobacillaceae* was significantly less abundant in IC/BPS. Missing significance in the Shannon and Simpson indices, with a concomitantly lower ace and Chao1 indices, indicated an overall lower microbial abundance in IC/BPS but no significant difference in diversity between the two groups.

On the other hand, there are also studies that could not demonstrate any correlation between interstitial cystitis and alterations in the urinary microbiome. In a study with premenopausal women performed by Meriwether et al. [68], the Chao1 and Simpson indices were similar in both urine and vaginal samples between IC/BPS and unaffected women. Furthermore, weighted and unweighted UniFrac analyses showed no differences between the groups. In contrast, there was a stronger correlation between the vaginal and urinary microbiome of women with interstitial cystitis, a fact which the researchers attribute to an altered function of the bladder epithelium. Bresler et al. [69] also failed to demonstrate clear significant differences in the urinary microbiome in the context of interstitial cystitis. Indicated by several alpha diversities, in his prospective, case-controlled study with 21 women with IC/BPS and 20 asymptomatic controls, and despite extending 16S rRNA analysis by a validated EQUC, control urine, IC/BPS vaginal swab and IC/BPS, urine did not differ significantly. Only at species level a marginally significant higher frequency of *Staphylococcus lugdunensis* and *Streptococcus agalactiae* in the urine of the control group and a tendency toward increased occurrence of *Escherichia coli* in IC/BPS patients could be demonstrated. Finally, even the results of Jacobs et al. [70] do not conclude that there is a connection between altered bladder microbiome and IC disease. The observed detection of *Streptococcus* only in the urine of affected participants, which also correlates with less severe symptoms, is difficult to explain. However, an important observation of this study is that there is a large discrepancy between urinated and catheterized samples, with up to 10,000-fold increased detected biomass in urinated samples. This confirms that voided urine does not accurately represent the bladder microbiota and that using voided samples to describe the bladder microbiome can lead to a significantly increased false positive rate.

An overview of the patterns of microbiome changes presented above for UUI, OAB, and IC/BPS is summarized in Table 1. It should be noted that the patterns described cannot represent a complete analysis of the bladder microbiome, as few studies have been performed to date, and the methods of microbiome determination do not yet follow uniform guidelines. Furthermore, the enormous individual, gender-dependent, and age-dependent variability of the bladder microbiome limits the ability to describe significant patterns.

### 3.3. No Microbiome-Based Etiology in Hunner Lesions

In approximately 10–20% of patients diagnosed with interstitial cystitis/bladder pain syndrome, so-called Hunner lesions (HLs) can be detected [71,72,73]. Visualized as a quite typical inflammatory reaction with a “circumscript”, reddened mucosal area in which small vessels are visible radiating to a central scar covered with a small central blood clot or fibrinous layer [19,74,75], the etiology of these lesions has never been definitively characterized [71]. As patients diagnosed with Hunner lesions appear to have more severe bladder-centric symptoms, are older, and show more severe symptomatology compared to patients without lesions [72,75,76], together with the observable inflammatory pathology, a correlation with specific bacterial species or microbial patterns associated within this subgroup in interstitial cystitis patients could be assumed. However, only one study has currently investigated possible changes between the microbiome in HL and non-HL interstitial cystitis patients [77]. Overall, 12 species have been shown to be differentially abundant between HL and non-HL patient samples; however, after applying Bonferroni adjustment, none of these species reach significantly differentially abundance between all HL vs. all non-HL patients and between female HL vs. non-HL patients. In contrast, in male subanalyses, a significant increase in *Negativicoccus succinivorans*, *Porphyromonas somerae* and *Mobiluncus curtisii* in HL patients, and in *Corynebacterium renale* in non-HL patients, could be determined. Further, Shannon diversity metrics showed a slightly significant higher alpha diversity among HL male patients than HL female patients, but not between HL and non-HL patients overall. Currently, it cannot be explained why these differences are found only between HL and non-HL men; however, it seems unlikely that these specific bacterial species are associated with HL pathogenesis in male patients only and not in females.

## 4. Conclusion—Is There Microbiome-Based Biomarker Potential in Interstitial Cystitis?

The detection of microbial colonization of the bladder, even in a healthy state, opens up a new and exciting field for science, especially in the investigation of etiological and diagnostic findings associated with urological diseases. Until a few years ago, the bladder was considered a sterile niche in the human body, and the detection of uropathogenic microorganisms was usually associated with the presence of urologic disorders. Interstitial cystitis/bladder pain syndrome is considered a diagnosis of exclusion because the etiological background is still unknown and no diagnostic markers are available to date. However, among many other factors, the microbiome has never been excluded as a possible regulator or trigger of this chronic disease. With the development of culture-independent metagenomic analyses by next-generation sequencing (NGS) or by enhanced quantitative urine culture (EQUC), alterations in the bladder microbiome associated with this disease have increasingly become the focus of current research. Shifts in the microbiome diversity and richness, as well as patterns of over- or under-representation of specific microorganisms, offer great potential to act as biomarkers for diagnosis, prognosis, risk profiling, and precision therapy, and would represent a milestone in IC/BPS research. However, the overall number of studies conducted in this field is still quite limited, and differences in the design of the study, sequencing technique, or level of organism identification make it difficult to compare the results obtained.

Another important factor that strongly influences the comparability of microbiome data received from different studies is the way of collecting urinary samples. To date, studies to determine the IC/BPS microbiome have analyzed both clean-captured midstream urine [60,67,68,69] and catheterized urine [61,66,70]. However, it should be noted that urethral and posturethral contamination, e.g., by vulvovaginal microbes in women or an overrepresentation of urethral microbes in men due to the anatomical feature of the longer urethra, must be considered in voided urine sampling. Thus, comparability between voided and catheterized microbiome samples is not meaningful, and for further studies, standardization of the collection technique as described, e.g., in [78], should be strived for.

In addition, the fact that the bladder microbiome is highly complex, individual, and age and sex dependent, as well as the fact that variability within a cohort may be greater than between affected and healthy persons, further complicates the disaggregation of possible patterns. Some study results suggest that the microbiome may play a protective role, for example, through higher levels of *Lactobacilli* in healthy individuals, and that the absence or reduced abundance of this genus in IC/BPS patients may have significance in the etiology of IC/BPS. A relationship between lower diversity or richness of the microbiome and IC/BPS disease has also been supposed. However, other studies have failed to uncover such relationships and do not currently allow us to identify a putative organism or urotype that clearly distinguishes the urinary microbiome of patients with IC/BPS from healthy controls and, thus, cannot actually act as a potential microbiome-based biomarker for diagnostic or prognostic purposes.

## Figures and Tables

**Table 1:** Patterns of species, genera and/or diversity microbiome changes for UUI (urge urinary incontinence), OAB (overactive bladder) and IC/BPS (interstitial cystitis/bladder pain syndrome) as demonstrated by cited studies.

Urological Disorder	Species/Genera and Diversity/Abundance Patterns	References
**UUI**	**Increased:** *Lactobacillus gasseri*, *Alteromonadaceae* spp., *Stenotrophomona*s spp., *Brevundimonas* spp., *Elizabethkingia* spp., *Methylobacterium* spp. **Decreased:** Microbial diversity, *Lactobacillus crispatus*, *Lactobacillus* sp.	[34,49,50]
**OAB**	**Increased:** *Sneathia, Staphylococcus, Proteus, Helcococcus, Gemella, Mycoplasma, Aerococcus* **Decreased:** *Prevotella, Dialister, Fusobacterium, Jonquetella, Campylobacter, Finegoldia, Anaerococcus, Pyramidobacter, Ureaplasma, Enterococcus, Novosphingobium, Lactococcus, Lactobacillus*	[57,58]
**IC/BPS**	**Increased:** *Lactobacillus johnsonii, Lactobacillus gasseri, Serratia, Brevibacterium, Porphyromonas, Citrobacter* **Decreased:** Number of detected genera, overall richness *Lactobacillus acidophilus*, *Corynebacterium* sp. *Senegalimassilia, Howardella, Gemella, Dialister, Moheibacter, Sphingobium, Fastidiosipila, Megasphaera, Thermovum, Mycobacterium, Prevotella, Gardnerella* **Exclusively shown in IC/BPS:** *Francisella tularensis, Mycoplasma hyorhinis, Helicobacter hepaticus, Clostridium perfringens, Candida dubliniensis, Enterococcus, Cronobacter, Escherichia coli, Proteus mirabilis, Pseudomonas aeruginosa* **Conflicting data:** *Atopobiom, species abundances and ecological diversity*	[60,61,66,67,68,69,70]

## Data Availability

Not applicable.
